# Racial/ethnic disparities in early‐onset colorectal cancer: implications for a racial/ethnic‐specific screening strategy

**DOI:** 10.1002/cam4.3811

**Published:** 2021-02-28

**Authors:** Ana R. Acuna‐Villaorduna, Juan Lin, Mimi Kim, Sanjay Goel

**Affiliations:** ^1^ Department of Medical Oncology Montefiore Medical Center Bronx NY USA; ^2^ Department of Medicine Albert Einstein College of Medicine Bronx NY USA; ^3^ Department of Epidemiology & Population Health Albert Einstein College of Medicine Bronx NY USA

**Keywords:** colorectal cancer, EO‐CRC, racial disparities

## Abstract

**Introduction:**

Early‐onset colorectal cancer (EO‐CRC) is a public health concern. Starting screening at 45 years has been considered, but there is discrepancy in the recommendations. Racial disparities in EO‐CRC incidence and survival are reported; however, racial/ethnic differences in EO‐CRC features that could inform a racial/ethnic‐tailored CRC screening strategy have not been reported. We compared features and survival among Non‐Hispanic White (NHW), Non‐Hispanic Black (NHB), and Hispanics with EO‐CRC.

**Methods:**

CRC patients from SEER 1973–2010 database were identified, and EO‐CRC was defined as CRC at <50 years. Clinical/pathological features and survival were compared between NHW, NHB, and Hispanics. Cancer‐specific survival (CSS) predictors were assessed in a multivariable Cox proportional hazard model.

**Results:**

Of 166,416 patients with CRC, 16,545 (9.9%) had EO‐CRC. The EO‐CRC frequencies in NHB and Hispanics were higher than NHW (12.7% vs. 16.5% vs. 8.7%, *p* < 0.001). EO‐CRC in NHB presents more frequently in females, with well/moderately differentiated, stage IV, and is less likely to present in locations targetable by sigmoidoscopy than NHW (54.6% vs. 67.7% OR:1.7, 95% *p* < 0.001). 5‐year CSS was lower in NHB (59.4% vs. 72.8%, HR: 1.7; 95% CI: 1.54–1.82) and Hispanics (66.4% vs. 72.8%, HR: 1.3; 95% CI: 1.16–1.39) than NHW. A regression model among patients with EO‐CRC showed that being NHB or Hispanic were independent predictors for cancer‐specific mortality, after adjusting for gender, grade, stage, and surgery.

**Conclusion:**

EO‐CRC is more likely in NHB and Hispanics. Racial disparities in clinical/pathological features and CSS between NHB and NHW/Hispanics were evidenced. A racial/ethnic specific screening strategy could be considered as an alternative for patients younger than 50 years.

## Graphical abstract

An alarming increase in the incidence of early‐onset colorectal cancer (EO‐CRC) has prompted updates in CRC screening guidelines; however, consensus has not been reached yet. Early‐onset colorectal cancer (EO‐CRC) presents more frequently in racial minority groups compared to Non‐Hispanic Whites (NHW). Compared to NHW/Hispanics, EO‐CRC in NHB presents more frequently in females, with a higher rate of well/moderately differentiated, advanced staged and right‐sided tumors. A race‐tailored CRC screening strategy including full colonoscopy in NHB and sigmoidoscopy in NHW and Hispanics could represent a cost‐effective method for screening in young population.

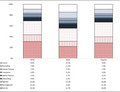

## 1 INTRODUCTION

During the last decade, the incidence of early‐onset colorectal cancer (EO‐CRC) has continuously risen at an annualized rate of 1.8%.^1^ It is estimated that 10.9% of all colon and 22.9% of all rectal cancers will be diagnosed in individuals younger than 50 years old by year 2023.^2^ As widespread CRC screening, starting at age 50, has proven effectiveness in decreasing the incidence of CRC in individuals 50 years or older; lowering the initiation age to target patients with EO‐CRC has been considered.^3^ The cost‐effectiveness of early CRC screening at different frequencies (every 10 or 15 years) and modalities (colonoscopy, sigmoidoscopy, fecal‐occult blood test) has been compared using microsimulation models; however, a consensus has not been reached yet. While the National Comprehensive Cancer Network support starting CRC screening at age 50, the American Cancer Society lowered the starting age to 45 and the American College of Gastroenterology suggests starting at age 45 only in Non‐Hispanic Blacks (NHBs). While the US Preventive Task Force (USPFT) has continued to recommend starting at age 50, it is currently evaluating lowering the starting age to 45 years. This was proposed in a draft recommendation statement in October 2020 and has not been confirmed yet.^4^, ^5^, ^6^, ^7^

Prior reports have evidenced racial disparities in the incidence and survival of patients with EO‐CRC. Compared to Non‐Hispanic Whites (NHWs), NHBs have a higher incidence rate (12.2 vs. 9.2 per 100,000)^8^ and lower 5‐year survival (62.8% vs. 68.6%).^8^ It is unknown whether this mortality difference is driven by race‐specific biologic differences or by inequity in access to care. Overall, it is known that EO‐CRC tumors tend to present more distally, with higher grade and stage at diagnosis; yet, with surprisingly better overall survival (OS) rates compared to CRC in patients 50 years or older.^9^, ^10^, ^11^, ^12^ The purpose of this study was to compare the characteristics and cancer‐specific survival of EO‐CRC by racial/ethnic groups using a large US population‐based cancer registry in order to identify differences that could help develop a race‐specific CRC screening strategy for patients younger than 50 years old.

## 2 METHODS

### 2.1 Data source

Adult patients with newly diagnosed CRC were identified using the Surveillance, Epidemiology and End Results (SEER) database from 1973 to 2010. SEER is a comprehensive database that collects information on cancer incidence, clinical/pathological characteristics, and survival from 20 population‐based cancer registries across the United States, representing approximately 28% of the population.

The SEER*Stat 8.15 software was interrogated to locate all cases of CRC adenocarcinoma by histology codes (8010, 8140, 8147, 8210, 8211, 8260, 8263, 8480, 8481, 8490) and anatomical primary site based on the International Classification of Diseases for Oncology–Third Edition (ICD‐O‐3) codes (C18.0–C21.1 from cecum to anal canal, C18.8 for overlapping lesion of colon, C18.9 for colon, not otherwise specified and C21.8 for overlapping lesion of rectum, anus, and anal canal. Only patients with available racial/ethnic groups in the SEER database reported as White (NHW), African American (NHB) and Hispanics were included. Duplicated entries (identified by Set ID number) were located and any second event; representing a second primary or recurrent CRC was excluded.

### 2.2 Variables and definitions

EO‐CRC was defined as CRC diagnosed in patients younger than 50 years old. Clinical characteristics included age at diagnosis (categorized as younger than 45 or between 45 and 49 years), gender and race/ethnicity, whereas pathology features included histological grade (categorized as well/moderate and poor/anaplastic), stage at initial diagnosis (non‐metastatic and metastatic), and tumor location (appendix to anus). We used tumor location to create two additional variables to classify tumors by sidedness (right‐sided: appendix to hepatic flexure and left‐sided: splenic flexure to anus) and to identify tumors targetable by sigmoidoscopy (from sigmoid colon to anus). Data regarding treatment available in SEER included surgical intervention and receipt of radiation. Overall survival (OS) was calculated using vital status and follow‐up time from diagnosis date and cancer‐specific survival (CSS) was calculated using the cause‐specific death classification variable provided in the SEER database.

### 2.3 Statistical analysis

EO‐CRC among patients with CRC in total and by race/ethnicity groups is reported as proportions and compared among groups using chi‐square. Bivariate associations between clinical/pathological characteristics and racial/ethnic group were tested using chi‐square for categorical and Kruskal–Wallis tests for non‐normally distributed continuous variables. When the overall difference between racial/ethnic groups was significant (*p* < 0.05), post‐hoc tests were performed and odds‐ratio with 95% confidence intervals for each comparison are reported. Kaplan–Meier survival analysis and log‐rank tests were used to assess and compare OS and CSS between racial/ethnic groups. To evaluate if race/ethnicity is an independent predictor of CSS, a multivariable Cox proportional hazard model was built including all variables. Given the lack of information about chemotherapy treatment, a second model including only patients diagnosed with stage I CRC was built. Statistical significance was determined by a two‐sided *p*‐value < 0.05. Analyses were conducted using Stata 15.1 (StataCorp) and SAS 9.4 (SAS Institute).

## 3 RESULTS

Of all CRC patients in the SEER cancer registry, EO‐CRC was seen in 16,545 (9.9%). The proportion of patients diagnosed with EO‐CRC within each racial/ethnic group differed significantly (NHW: 8.7%, NHB: 12.7%, and Hispanics: 16.5%) (Table 1). EO‐CRC was more likely diagnosed among NHB (OR: 1.53, 95% CI: 1.45–1.59, *p* < 0.001) and Hispanics (OR:2.06, 95% CI:1.98–2.17, *p* < 0.001) than NHW.

**TABLE 1:** Characteristics of patients with early‐onset colorectal cancer from the surveillance, epidemiology, and end‐result database by racial/ethnic groups

EO‐CRC *n* = 16,545	NHW *n* = 11,320	NHB *n* = 2553	Hispanic *n* = 2672	*p*‐values for differences across groups	
				NHW versus NHB	NHW versus Hispanic
EO‐CRC/All CRC	8.7%	12.7%	16.5%	<0.001	<0.001
Age, median (IQ)	45 (40–47)	45 (40–47)	44 (38–47)	0.75	<0.001
Age groups					
Less than 45	48.6%	49.9%	56.1%	0.24	<0.001
From 45–49	51.4%	50.1%	43.9%		
Gender					
Female	46.5%	51.9%	46.9%	<0.001	0.67
Male	53.5%	48.1%	53.1%		
Grade					
Well/moderate	79%	82%	79.8%	0.002	0.43
Poor/anaplastic	21%	18%	20.2%		
Stage					
I–III	76.3%	70.7%	74.7%	<0.001	0.08
IV	23.7%	29.3%	25.3%		
Sidedness					
Right‐sided	21.2%	32.1%	22.3%	<0.001	0.24
Left‐sided	78.8%	67.9%	77.7%		

Note

±Early‐onset Colorectal Cancer was defined as diagnosis before age 50.

CI, Confidence Interval; IQ, 25%–75% interquartile range; NHB, Non‐Hispanic Black; NHW, Non‐Hispanic White.

### 3.1 Characteristics by racial/ethnic groups in EO‐CRC

The proportion of patients diagnosed at ages younger than 45 versus 45 to 49 years differed between NHW, NHB, and Hispanics (48.6% vs. 49.9% vs. 56.1%, *p* < 0.001), respectively (Table 1). Compared to NHW, Hispanics were more likely to be diagnosed before 45 years of age (OR: 1.35, 95% CI: 1.24–1.47, *p* < 0.001), whereas no difference was seen between NHW and NHB (OR: 1.1, 95% CI: 0.97–1.15, *p* = 0.24).

Gender, grade, stage, and sidedness were similar between NHW and Hispanics but differed between NHW and NHB (Table 1). NHB were more likely female (OR: 1.24; 95% CI: 1.14–1.36), had well/moderately differentiated tumors (OR: 1.20, 95% CI: 1.07–1.35), metastatic disease at diagnosis (OR: 1.34; 95% CI: 1.21–1.47) and right‐sided tumors (OR: 1.75, 95% CI: 1.59–1.94) compared to NHW.

**FIGURE 1:**
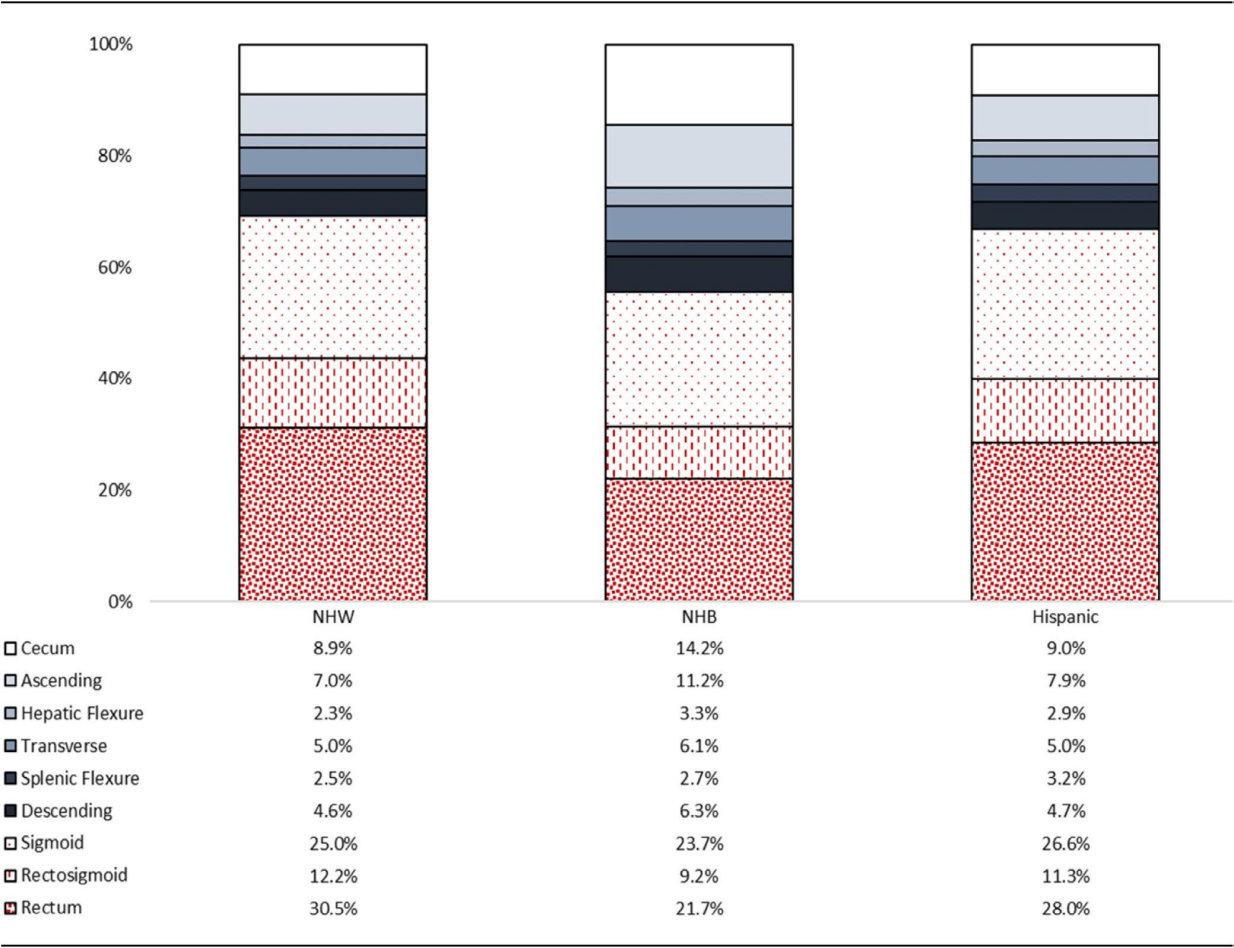
Tumor site distribution in patients with early‐onset colorectal cancer from the surveillance, epidemiology, and end‐result database by racial/ethnic groups. Early‐onset colorectal cancer was defined as diagnosis before age 50 (NHB, Non‐Hispanic Black; NHW, Non‐Hispanic White; red‐color bars represent lesions targetable by sigmoidoscopy)

Regarding tumor location distribution, no differences were noted between NHW and Hispanics, while the tumor location pattern varied in NHBs compared to the other racial/ethnic groups. The rectum was the most common location in NHW (30.5%) and Hispanics (28%), whereas it accounted for 21.7% in NHB. In contrast, 25.4% of tumors in NHB were in the cecum and ascending colon, while only 15.9% tumors in NHW and 16.9% in Hispanics were found in these locations. Among 72.3% and 70.6% of tumors that were left‐sided in NHW and Hispanics, respectively; 67.7% and 65.9% were in a location targetable by sigmoidoscopy. In NHB, 60.9% were left‐sided and 54.6% were targetable by sigmoidoscopy (Figure 1). Tumors targetable by sigmoidoscopy were 73% more frequent in NHB than NHW (OR: 1.7, 95% CI: 1.58–1.89, *p* < 0.001).

### 3.2 Access to treatment by racial/ethnic group in EO‐CRC

Among patients with non‐metastatic EO‐CRC, surgery was lower in NHB (OR: 0.52, 95% CI: 0.39–0.68, *p* < 0.001) and Hispanics (OR: 0.51, 95% CI: 0.38–0.66, *p* < 0.001) compared to NHW. Similarly, among 5242 patients diagnosed with EO‐CRC rectal cancer, the frequency of radiation was significantly lower in NHB compared to NHW (61.3% vs. 66.9%, OR: 0.78, 95% CI: 0.65–0.94) whereas no difference was seen among NHW and Hispanics.

### 3.3 Mortality by racial/ethnic group and mortality predictors in EO‐CRC

**FIGURE 2:**
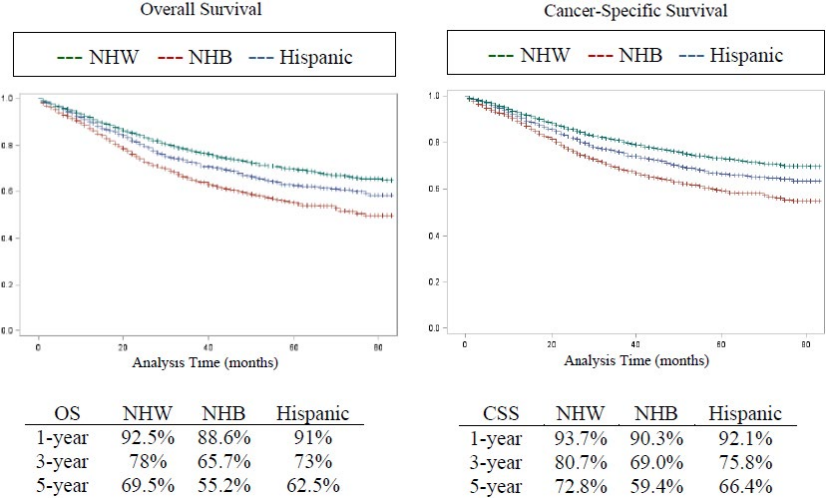
Kaplan–Meier survival analysis stratified by racial/ethnic groups among patients with early‐onset colorectal cancer from the surveillance, epidemiology, and end‐result Database. Early‐onset colorectal cancer was defined as diagnosis before age 50 (CSS, Cancer‐specific survival; NHB, Non‐Hispanic Black; NHW, Non‐Hispanic White; OS, Overall survival)

No statistically significant differences in 1‐year OS or CSS were seen among racial/ethnic groups, whereas OS and CSS disparities were evidenced at 5‐years. Compared to NHW, 5‐year CSS was lower in NHB (59.4% vs. 72.8% HR: 1.7; 95% CI: 1.54–1.82, *p* < 0.001) and Hispanics (66.4% vs. 72.8%; HR: 1.3; 95% CI: 1.16–1.39; *p* < 0.001) (Figure 2).

A multivariable Cox regression model in patients with EO‐CRC demonstrated that racial/ethnic group was independently associated with an increased risk of cancer‐related death, after adjusting for gender, stage, grade, sidedness, surgery, and radiation. The mortality risk was 53% higher in NHB (HR: 1.53, 95% CI: 1.39–1.69, *p* < 0.001) and 19% higher in Hispanics (HR: 1.19, 95% CI: 1.08–1.33, *p* = 0.001), compared to NHW. A second model, including only patients diagnosed with stage I, showed a non‐significant association between racial/ethnic groups and cancer‐specific death after adjusting for gender, grade, sidedness, surgery, and radiation (Table 2).

**TABLE 2:** Results of multivariable cox regression of cancer‐specific survival in patients with early‐onset colorectal cancer from the surveillance, epidemiology and end‐result database

	Model 1: All stages (*n* = 16,545)			Model 2: Stage I only (*n* = 3482)		
	HR	95% CI	*p‐*value	HR	95% CI	*p‐*value
Race						
NHW	1			1		
NHB	1.53	1.39–1.69	<0.001	1.40	0.74–2.64	0.31
Hispanic	1.19	1.08–1.33	0.001	0.87	0.39–1.93	0.72
Gender						
Female	1			1		
Male	0.86	0.79–0.93	<0.001	0.36	0.20–0.61	<0.001
Grade						
Well	1			1		
Moderate	0.92	0.77–1.09	0.33	0.87	0.42–1.78	0.69
Poor	1.60	1.47–1.75	<0.001	1.42	0.69–2.91	0.34
Anaplastic	1.84	1.45–2.34	<0.001	N/A		
Sidedness						
Left‐sided	1			1		
Right‐sided	1.33	1.21–1.46	<0.001	0.81	0.34–1.94	0.81
Surgery						
No	1			1		
Yes	0.38	0.34–0.42	<0.001	0.13	0.07–0.24	<0.001
Radiation						
No	1			1		
Yes	1.00	0.91–1.10	0.96	2.86	1.70–4.80	<0.001
Stage						
I	1					
II	2.51	1.90–3.30	<0.001			
III	5.62	4.37–7.23	<0.001			
IV	31.65	24.75–40.47	<0.001			

Note

Early‐onset Colorectal Cancer was defined as diagnosis before age 50.

Abbreviations: CI, Confidence Interval; NHB, Non‐Hispanic Black; NHW, Non‐Hispanic White.

### 3.4 Survival by stage and by sidedness among racial/ethnic groups

Survival analyses were performed after stratifying by stage at diagnosis and by sidedness. Unadjusted 1‐year CSS at all stages was similar among all racial/ethnic groups. In 5 years, no significant difference was seen in patients with stage I, while CSS was lower in NHB compared to NHW with stage II disease; and CSS was lower in NHB and Hispanics compared to NHW with stage III and IV diseases (Table 3).

**TABLE 3:** Cancer‐specific survival stratified by stage and sidedness among patients with early‐onset colorectal cancer from the surveillance, epidemiology, and end‐result database

Stage	1‐year CSS			5‐year CSS		
	NHW	NHB	Hispanic	NHW	NHB	Hispanic
I	99.5%	98.8%	99.3%	96.4%	94.8%	94.5%
II	99.2%	98.2%	98.8%	90.7%	81%	90.5%
III	98.1%	97%	97.2%	80.3%	68.4%	71.2%
IV	77%	72.2%	74.2%	22.3%	12.3%	15.5%
Sidedness						
Right‐sided	89.9%	89.1%	93%	69.9%	59.2%	71.2%
Left‐sided	94.9%	91.6%	92.9%	73.7%	60.2%	65.9%

Note

Early‐onset Colorectal Cancer was defined as diagnosis before age 50.

Abbreviations: NHW, Non‐Hispanic White; NHB, Non‐Hispanic Black; CSS, Cancer‐specific survival

Unadjusted 1‐year CSS in right‐sided and left‐sided tumors was similar among all racial/ethnic groups. Compared to NHW, NHB had lower CSS in right‐sided (69.9% vs. 59.2) and left‐sided tumors (73.7% vs. 60.2%) at 5 years (Table 3).

## 4 DISCUSSION

This study highlighted racial disparities in the frequency, clinical/pathological characteristics, and survival of patients with EO‐CRC. Consistent with prior reports, we also observed an EO‐CRC frequency of 9.9%;^11^, ^13^, ^14^ however, it varied by racial/ethnic group with statistically significantly higher frequencies seen in minorities. The odds of EO‐CRC were 100% higher in Hispanics and 50% higher in NHB compared to NHW. As estimated by US‐based population projections, the proportion of racial/ethnic minorities will continue to expand in the next decades and it is expected to overcome NHWs in frequency by 2060.^15^ Thus, increases in EO‐CRC cases are expected unless effective public health preventive measures are implemented. Based on this, we believe that early CRC screening is necessary and should not be restricted to NHB only. Accordingly, we support the most recent USPSTF draft recommendations to offer early screening to all patients.^16^

Consistent with differences reported among CRC patients diagnosed at 50 years or older, significant racial disparities were demonstrated in EO‐CRC and identified NHB as a group that suffers disproportionally compared to others. Besides presenting more frequently with metastatic disease compared to other racial/ethnic groups, our data showed that NHB are more likely to have right‐sided tumors than NHW or Hispanics. While nearly two‐thirds of tumors in NHW or Hispanics are localized between the sigmoid colon and rectum, only half of NHBs with EO‐CRC presented with a tumor in these locations. This is an interesting and critical finding that allow us to hypothesize that a racial/ethnic group‐specific CRC screening program, in which NHW and Hispanics younger than 50 could be evaluated with sigmoidoscopy, whereas full colonoscopy could be limited to NHBs, could favorably balance diagnostic yield versus procedure costs and complications. Further studies assessing the cost‐effectiveness of a racial/ethnic‐tailored CRC screening in patients younger than 50 are warranted.

Another current discrepancy point is regarding the age to start early CRC screening. While 45 years has already been recommended by some societies, starting at age 40 has also been contemplated.^5^, ^6^, ^7^ Based in our analysis, 44% of all EO‐CRC in Hispanics and 50% in NHW and NHB are diagnosed between 45–49 years old. Lowering the cutoff age to 40 years would allow the identification of 70% of all EO‐CRC in Hispanics and more than 75% in NHW and NHB. Whether 40 or 45 years is the most appropriate age to start CRC screening goes beyond the scope of this study as it should factor in additional variables such as procedure costs and complications which are not collected in the SEER database. Cost‐effectiveness analysis evaluating a racial/ethnic‐ specific screening program starting at 40 and 45 years old should also be considered.

Unfavorable outcomes among NHBs are recognized across several cancer types including CRC;^17^, ^18^ however, it remains unclear whether this is determined by tumor biological differences or delays in access to care. While molecular differences across racial/ethnic groups are well‐established, it seems unlikely that it would solely explain unfavorable outcomes in NHBs as only *BRAF* mutation has shown prognostic value and it does not seem to differ between NHW and NHB.^19^ In contrast, microsatellite instability (MSI), which confers favorable outcomes in stage II tumors, presents more frequently in right‐sided tumors.^20^ As right‐sided tumors are more common in NHB, they would be expected to have better, not worse prognosis. For the latter, delays in access to care, which could be driven by socio‐economic or cultural barries, have also been considered broadly in prior studies. Delays in adjuvant chemotherapy and its impact in mortality and disease recurrence has been analyzed in multiple retrospective studies with heterogeneous results.^21^, ^22^, ^23^ Discrepancies might be explained by several limitations including an arbitrary cut‐off selection to define treatment as delayeddifferences in the patient population (colorectal vs. colon vs. rectal; stage II/III vs. stage III, chemotherapy regimen:FU alone vs. FOLFOX and treatment duration: 3 vs. 6 months, among others) and the lack of important variables such as molecular markers in regression models.

In this study, no differences were evidenced in 1‐year survival across racial/ethnic groups; however, unfavorable outcomes became apparent in minorities at 5 years. We believe this late‐onset differential is suggestive of higher recurrence rates in minorities compared to NHW, which has been reported in other cancer‐registry based studies. Whether this disparity is driven by biological factors or by access to care remains unclear. In this regard, SEER data is limited to make a strong conclusion; however, we hypothesize that access to care plays a pivotal role to explain this differential for two reasons. First, lower surgery rates were evidenced among NHB and Hispanic patient with non‐metastatic disease and lower radiation rates were seen in NHB compared to NHW patients with rectal cancer. Second, while the racial/ethnic group was an independent predictor of cancer‐specific mortality in the multivariable analysis, the association did not hold when the analysis was restricted to patients with stage I only. Since management of patients with stage I disease entails surgery but does not require subsequent, regular visits as chemotherapy treatment does, we consider that access to care would mainly affect the availability of medical therapy, including chemotherapy, in the management of patients with stage II‐IV disease. Further studies are required to test this hypothesis.

This study has limitations that should be considered. SEER‐database lacks information about prognostic molecular features and systemic treatment which has proven to improve survival in high‐risk stage II and stage III CRC patients. Also, information regarding treatment is limited to describing whether surgery and/or radiation were given to the patient at any point; whereas information about chemotherapy regimens, the use of biologics, immunotherapy, participation in clinical trials is not provided. The lack of these information impairs the possibility of determining whether biological features or access to treatment are responsible for survival disparities across racial/ethnic groups.

In summary, racial disparities are evident in the frequency, clinical/pathological features, and outcomes of patients with EO‐CRC. Based on differences in location between NHB and NHW/Hispanics, we do not believe a “one size fits all approach” is the most cost‐efficient CRC screening strategy. Instead, a race/ethnic group‐specific CRC screening program, in which NHW and Hispanics younger than 50 would be evaluated with sigmoidoscopy, whereas full colonoscopy would be limited to NHBs, could represent a method that would favorably balance diagnosis yield versus procedure costs and complications. Economic studies evaluating a race/ethnic group specific strategy are warranted.

To the best of our knowledge, this study is the first to hypothesize that a race‐specific early CRC screening strategy could be considered as an alternative for patients younger than 50 years old. A cost‐effectiveness evaluation of a race‐specific early CRC strategy that would limit full colonoscopies to NHB; while favoring interventions to target the left colon such as sigmoidoscopies in Hispanics and NHW is warranted.

## CONFLICT OF INTEREST

The authors report no conflict of interest.

## Data Availability

All data underlying this study is available after request at SEER.gov.
